# Slow Transition Path Times Reveal a Complex Folding Barrier in a Designed Protein

**DOI:** 10.3389/fchem.2020.587824

**Published:** 2020-12-07

**Authors:** Alexander Mehlich, Jie Fang, Benjamin Pelz, Hongbin Li, Johannes Stigler

**Affiliations:** ^1^Physics Department E22, Technische Universität München, Garching, Germany; ^2^Department of Chemistry, University of British Columbia, Vancouver, BC, Canada; ^3^Gene Center Munich, Ludwig-Maximilians-Universität München, Munich, Germany

**Keywords:** protein folding, transition path analysis, artificial protein, transition state barrier, roughness, Kramers rate theory

## Abstract

*De-novo* designed proteins have received wide interest as potential platforms for nano-engineering and biomedicine. While much work is being done in the design of thermodynamically stable proteins, the folding process of artificially designed proteins is not well-studied. Here we used single-molecule force spectroscopy by optical tweezers to study the folding of ROSS, a *de-novo* designed 2x2 Rossmann fold. We measured a barrier crossing time in the millisecond range, much slower than what has been reported for other systems. While long transition times can be explained by barrier roughness or slow diffusion, we show that isotropic roughness cannot explain the measured transition path time distribution. Instead, this study shows that the slow barrier crossing of ROSS is caused by the population of three short-lived high-energy intermediates. In addition, we identify incomplete and off-pathway folding events with different barrier crossing dynamics. Our results hint at the presence of a complex transition barrier that may be a common feature of many artificially designed proteins.

## Introduction

The protein-folding problem has fascinated scientists for more than half a century (Anfinsen et al., 1961). To learn and understand more about the reaction that takes place when an unfolded polypeptide chain tries to “find” its correctly folded protein structure, the concept of energy landscapes is a powerful theoretical framework (Onuchic et al., 1997). Within this framework, three key parameters govern the typical timescales of an observed reaction: the height of a free energy barrier, which needs to be overcome, the curvature or stiffness at the top of that barrier, and the diffusion coefficient (Hänggi et al., 1990).

Experimentally, various techniques have been used to verify the applicability of energy landscape theory to protein folding. Besides single-molecule Förster resonance energy transfer (smFRET) (Borgia et al., 2012; Chung et al., 2012; Soranno et al., 2012; Chung and Eaton, 2013), another well-established tool to measure the folding of individual proteins is single-molecule force spectroscopy (SMFS), where force can act as both a denaturant and a readout of protein conformational changes (Rief et al., 1997; Junker et al., 2009; Gebhardt et al., 2010; Yu et al., 2012). In the case of optical tweezers, specific attachment strategies and stable setups have provided access to a large range of timescales and enabled measurements of single molecules over tens of minutes (Stigler et al., 2011; Rognoni et al., 2014) at a temporal resolution to resolve timescales of only a few tens of microseconds (Žoldák et al., 2013; Neupane et al., 2016). Recent studies have shown that with this technique it is possible to extract and evaluate transition paths [i.e., the rare events of actual barrier crossing, which allow the direct estimation of diffusion coefficients or the determination of transition path velocities (Neupane et al., 2017, 2018)]. In addition, the theoretical foundation used to analyze and correctly interpret experimentally measured transition paths is constantly improving (Hummer, 2004; Cossio et al., 2015, 2018; Covino et al., 2019).

A complementary approach to understanding protein folding tackles the problem from the opposite end (i.e., by *de-novo* designing protein sequences that fold into the desired shape). Because of their far-reaching potential in pharmaceutical applications, these artificial proteins have received wide interest from science and industry (Kuhlman and Bradley, 2019). In general, the *de-novo* design of proteins is an optimization problem where candidate sequences of amino acids are scored according to an energy model and a target fold. The optimization often comes at a high computational cost, as different side-chain packing can yield the same general fold, but widely different energy scores. While designers have been very successful in creating thermodynamically stable proteins as building blocks, there is little known about how these proteins compare to natural counterparts in terms of folding or conformational dynamics.

In this study, we characterize the timescales of folding and barrier crossing of one of the first fully designed artificial proteins with a topology that is abundantly found in nature, the 2x2 Rossmann fold, which can serve as a scaffold for designed enzymes (Koga et al., 2012).

## Materials and Methods

### Protein Expression and Sample Preparation

The sequence of the 2x2 Rossmann fold protein (ROSS) was based on the Di-II-10 sequence of Koga et al. (2012) where the part of the designed protein comprises 100 amino acids (bold in sequences below). We modified this sequence with additional n- and c-terminal cysteines and a c-terminal His_6_-tag. The variant S49Cc lacked the n-terminal cysteine and harbored two cysteines at position 49 and the c-terminus. Protein expression and purification were performed as described previously (Fang et al., 2013).

ROSS: MACK **MLLYVLIISN DKKLIEEARK MAEKANLELR TVKTEDELKK YLEEFRKESQ NIKVLILVSN DEELDKAKEL AQKMEIDVRT RKVTSPDEAK RWIKEFSEEG** GSKCLE HHHHHH.

S49Cc: MASKGS **MLLYVLIISN DKKLIEEARK MAEKANLELR TVKTEDELKK YLEEFRKE***C***Q NIKVLILVSN DEELDKAKEL AQKMEIDVRT RKVTSPDEAK RWIKEFSEEG** GSSGKCLE HHHHHH.

Bead-DNA-protein-bead-DNA dumbbells were generated as described previously (Mehlich et al., 2015). In short, maleimide-modified oligonucleotides were attached to the cysteines of the purified protein via a disulfide bond to form a protein-oligonucleotide construct. Next, biotin or digoxigenin modified DNA handles equipped with a single-stranded overhang complementary to the maleimide-modified oligonucleotides on their 3′ end were hybridized to this protein-oligonucleotide. The resulting DNA-protein-DNA hybrid was then bound to streptavidin or anti-digoxigenin functionalized 1 μm diameter silica beads for optical tweezers measurements.

### Optical Trap Setup and Measurement Modes

A custom-built dual optical trap setup with back-focal plane detection was used for all force spectroscopic measurements (von Hansen et al., 2012). The two measurement modes comprise either stretch-relax cycles in constant-velocity mode or measurements at constant trap distances in passive-mode.

Before recording, signals were filtered with an eighth order Butterworth filter with a 3 dB-frequency set at 100 kHz. Data were acquired at 200 kHz. The stiffness of each trap was typically set to about 0.2 pN/nm. Constant-velocity measurements were performed at 500 nm/s.

### Polymer Models and Length Coordinates

To model the force compliance of the polymer linkers that connect the protein to the beads we used previously published polymer models (Bustamante et al., 1994; Wang et al., 1997). Upon stretching and while the protein remains folded, the mechanical response of a trapping construct is dominated by stretching of the DNA handles which were modeled using an extensible Worm-Like Chain (eWLC) model (Wang et al., 1997)

(1) $$\begin{array}{l} \begin{array}{l} F\left(\xi_{D}\right)=\frac{k_{\text{B}}T}{p_{D}}\left(\frac{1}{4}{\left(1-\frac{\xi_{D}}{L_{D}}+\frac{F}{K}\right)}^{-2}-\frac{1}{4}+\frac{\xi_{D}}{L_{D}}-\frac{F}{K}\right) \end{array} \end{array}$$

with DNA extension ξ_*D*_, force *F*, thermal energy *k*_B_*T*, DNA persistence length *p*_*D*_, DNA contour length *L*_*D*_, and stretch modulus *K*.

The additional force-extension of unfolded protein was modeled using a Worm-Like Chain (WLC) model in series with the above response of the DNA handles;

(2) $$\begin{array}{l} \begin{array}{l} F\left(\xi_{p}\right)=\frac{k_{\text{B}}T}{p_{p}}\left(\frac{1}{4}{\left(1-\frac{\xi_{p}}{L_{p}}\right)}^{-2}-\frac{1}{4}+\frac{\xi_{p}}{L_{p}}\right) \end{array} \end{array}$$

where ξ_*p*_ is the additional extension of the unfolded amino acid chain of the protein, *p*_*p*_ is the protein persistence length, and *L*_*p*_ is the protein contour length.

To fit our experimental data, *p*_*p*_ was fixed at 0.7 nm. Typical values for the DNA fits were *p*_*D*_ ≈ 31 nm, *L*_*D*_ ≈ 364 nm, and *K* ≈ 200 pN. Experiments were conducted at a temperature of *T* ≈ 298 K.

Instead of the molecular extension ξ_*p*_, which depends on the applied force, we used the force-independent contour length of the unfolded polypeptide *L*_*p*_ as a reaction coordinate. At a trap distance *d*, omitting the comparatively small extension of the folded protein, the extension of the unfolded polypeptide is given by $\xi_{p}=d-\frac{F}{k_{c}}-\xi_{D}\left(F\right)$, where $k_{c}={\left(k_{1}^{-1}+k_{2}^{-1}\right)}^{-1}$ is the combined spring constant of the two beads and $x=\frac{F}{k_{c}}$ is the combined bead deflection (see Supplementary Figure 1). The contour length *L*_*p*_ was then obtained from ξ_*p*_ using Equation (2). This conversion allowed us to compare events at different forces in a more straightforward manner (Puchner et al., 2008).

Integrals over the polymer models were used to convert energy profiles at different force biases as described (Ramm et al., 2014).

### Experimental Rate Constants and Lifetime Distributions

Rate constants for unfolding and refolding from constant-velocity experiments were determined using cycles with 500 nm/s pulling speed and applying the method by Oberbarnscheidt et al. (2009).

Folding/unfolding rate constants from passive-mode measurements were obtained from hidden Markov models (HMMs) as described previously (Geier et al., 2007; Stigler et al., 2011; Stigler and Rief, 2012). In brief, HMMs were used to determine the likeliest sequence of hidden states that describes the observed trajectory of bead deflections. The lifetimes for each detected state were well-described by a single exponential distribution. Transition rate constants between states *i* and *j* were then determined using $k_{ij}=N_{ij}/\left(\tau_{i}\cdot \underset{k}{\sum }N_{ik}\right)$, where τ_*i*_ is the average lifetime of state *i* and *N*_*ij*_ is the number of detected transitions between states *i* and *j*.

Force-dependent folding/unfolding rate constants were fitted to a model (Equation (S6), see Supplementary Information) that incorporates the energetic contributions of the non-linear polymer linkers to yield transition state positions and extrapolate force-free transition rate constants (Schlierf et al., 2007; Stigler et al., 2011).

### The Relation Between Rates and Shape Parameters of an Energy Landscape

A relation between the transition rate *k*_*ij*_ from state *i* to *j* and the shape of the barrier with a height Δ*G*^*i*TS^ separating these two states is given by Kramers' rate theory in the Smoluchowski limit [i.e., the overdamped case where ${\left(D\cdot \beta \right)}^{-1}\gg \omega_{\text{TS}}$ (Kramers, 1940; Hänggi et al., 1990)]:

(3) $$\begin{array}{l} \begin{array}{l} k_{ij}=\frac{D\cdot \beta \cdot \omega_{i}\cdot \omega_{\text{TS}}}{2\pi }\cdot \exp\left(-\beta \cdot \Delta G^{i\text{TS}}\right) \end{array} \end{array}$$

Here, *D* is the diffusion coefficient, β is the inverse thermal energy ($\beta^{-1}=k_{\text{B}}T$), and ω_*i*_ and ω_TS_ represent the oscillation frequencies around the minimum of state *i* and around the barrier top of the transition state which needs to be passed to reach state *j*. We used this relation for barrier height reconstruction or expected rate calculation under the assumption of symmetric and harmonic barrier shapes. This assumption implies that ω_*i*_ = ω_TS_ and $\omega_{\text{TS}}^{2}$ = $\left|\partial_{x}^{2}G\right|$ = $4\cdot \frac{{\Delta G}^{i\text{TS}}}{{\left({\Delta x}^{\text{i}\text{TS}}\right)}^{2}}$, where *x*^*i*TS^ is the distance between a state *i* and its adjoining transition state TS (Mehlich, 2018).

### Deconvolution of Equilibrium Distributions

For deconvolution (i.e., the removal of thermal noise-broadening) of measured bead deflection distributions *P*_msmt_(*x*), we used the same algorithm as described in Ramm et al. (2014). In brief, we numerically minimized the function

(4) $$\begin{array}{r} O={\langle \left|\ln\left({\hat{P}}_{\text{prot}}\left(x\right)⊗\Psi_{x}\left(x\right)\right)-\ln\left(P_{\text{msmt}}\left(x\right)\right)\right|\rangle }_{x}+\lambda \cdot \\ \underset{i}{\sum }{\left[\beta \cdot \partial_{x}^{2}\;\hat{G}\left(x\right){|}_{{\hat{x}}_{i}}\right]}^{2} \end{array}$$

where ⊗ represents the convolution operator and Ψ_*x*_(*x*) is the force-dependent point-spread function (PSF) to estimate the true probability distribution ${\hat{P}}_{\text{prot}}\left(x\right)=\;\exp\left(-\beta \cdot \hat{G}\left(x\right)\right)$. The only unknown quantity was the deconvolved energy landscape *Ĝ*(*x*) of the protein. The smoothing penalty parameter was set to λ = 10^−2^nm^4^.

The shape of the PSF was determined from equilibrium thermodynamics calculations (see Ramm et al., 2014 for a detailed description). In brief, at a given trap distance *d*, the mechanical free energy of stretching the polymer linkers and displacing the beads from their traps is

(5) $$\begin{array}{l} \begin{array}{l} H\left(x\right)=\int_{0}^{d-x}F_{D+p}\left(\xi \right)d\xi +\frac{1}{2}k_{c}x^{2}, \end{array} \end{array}$$

where *F*_*D*+*p*_(ξ) is the inverse of ξ_*D*_(*F*) + ξ_*p*_(*F*) (Equations (1), (2)). The theoretical PSF of deflection values for a given trap distance and a given unfolded contour length was then determined by

(6) $$\begin{array}{l} \begin{array}{c} \Psi_{x}(x)=\frac{e^{-\beta H\left(x\right)}}{\int e^{-\beta H\left(x\right)}dx} \end{array} \end{array}$$

Because of the nonlinearity of the eWLC and WLC linkers, the theoretical PSF is asymmetric and well-approximated by a skewed Gaussian distribution (See Azzalini, 1985 for a description of the skewed Gaussian distribution). Supplementary Figure 2 shows the theoretical PSF in contour-length space for the states N and U, at typical forces in passive-mode experiments and in stretch-relax experiments, which we parameterized by a scale parameter σ and a skewness parameter γ. Because the force is not constant in passive-mode experiments, σ = σ(*x*) and γ = γ(*x*) are position-dependent (Gebhardt et al., 2010). In our analysis, σ(*x*) and γ(*x*) were determined by linear interpolation between values obtained from fits to the distribution of the folded state and the unfolded state.

### Determination of Transition Path Time

Hidden Markov models (HMM) were used to identify state positions, state occupancies, and dwell times for transitions between folded (N) and unfolded (U) states of ROSS as described elsewhere (Stigler et al., 2011). To assess the actual duration of these identified transitions, we applied a 3-state model where the two outer states were fixed at the identified N and U positions and a third obligatory intermediate state T was introduces and fixed right in the middle between N and U, similar to an approach already used elsewhere (Chung et al., 2012; Sturzenegger et al., 2018). The dwell times measured for T were interpreted as the required transition path time to cross the barrier between the folded and unfolded state.

For validation, the HMM method was also applied with, instead of one central intermediate T, two intermediates at the positions of I_1_ and I_2_. Here, transition path times were derived as the sum of the dwell times spent within I_1_ and I_2_ along each transition between N and U. The two methods resulted in practically identical estimates of the transition path time (TPT) from passive-mode measurements ($\langle \tau^{{\text{I}}_{1}+{\text{I}}_{2}}\rangle$ = 1.6 ± 0.1 ms and 〈τ^T^〉 = 1.5 ± 0.1 ms). Supplementary Figures 3A,B shows a comparison between results obtained from both methods. We note that the HMM method does not strictly prohibit boundary re-crossing. When imposing absorbing boundaries, we found that the detected transition paths often excluded stretches that lie visibly on a path (Supplementary Figure 3C) and the corresponding TPTs were consequently underestimated. This underestimation was due to random bead fluctuation noise that did not reflect changes in the molecular length and was especially prominent at high bandwidth. Filtering to lower bandwidth suppressed this noise and resulted in TPTs that agreed with both the HMM method (see above) and the method of transition averaging (see below).

Alternatively, transition path times were determined by selecting individual transitions and fitting the high-bandwidth bead relaxation data to a sigmoidal equation $f\left(t\right)=A/\left(1+\exp\left(\frac{t-t_{0}}{\tau_{s}}\right)\right)$, to determine the time *t*_0_ at which a transition occurred. The transitions were then aligned according to their *t*_0_ values and averaged. The characteristic timescale τ_*s*_ of the transition was then obtained from a sigmoidal fit to the aligned and averaged data (Supplementary Figures 4B,C). To be able to compare the characteristic timescales of the obligatory intermediate model and the sigmoidal model, we performed simulations of transitions between two states with an obligatory intermediate in the center (Supplementary Figure 4D, inset). The duration of the intermediate dwell was chosen from an exponential distribution with a time constant τ. Mimicking experiments, the transitions were then aligned, averaged, and fitted to a sigmoidal equation to determine the characteristic timescale of the sigmoidal model τ_*s*_. A conversion factor between the two timescales τ and τ_*s*_ was then obtained by repeating the simulation for different intermediate durations (Supplementary Figure 4E).

### Transition Path Time Analysis

An approximation for the average transition path time 〈τ_TP_〉 which is required to cross a harmonic barrier of height $\Delta G^{‡}>2\;k_{\text{B}}T$ by one-dimensional diffusion is given by Chung et al. (2009):

(7) $$\begin{array}{l} \begin{array}{l} \langle \tau_{\text{TP}}\rangle \approx \frac{\ln\left(2e^{\gamma_{E}}\cdot \beta \cdot \Delta G^{‡}\right)}{\beta \cdot D\cdot \omega_{\text{TS}}^{2}} \end{array} \end{array}$$

where γ_*E*_ ≈ 0.577 is Euler's constant.

The following model approximates the cumulative distribution function of transition path times over a harmonic barrier (Zhang et al., 2007; Chaudhury and Makarov, 2010):

(8) $$\begin{array}{l} \begin{array}{l} P\left(\tau_{\text{TP}}\right)\approx \int_{0}^{\tau_{\text{TP}}}\frac{\omega_{K}\cdot \sqrt{\beta \cdot \Delta G^{‡}}}{1-\mathrm{erf}\left(\sqrt{\beta \cdot \Delta G^{‡}}\right)}\cdot \frac{\exp\left[-\beta \cdot \Delta G^{‡}\cdot \coth\left(\omega_{K}\cdot \frac{\tau }{2}\right)\right]}{\sinh\left(\omega_{K}\cdot \frac{\tau }{2}\right)\cdot \sqrt{2\pi \cdot sinh\left(\omega_{K}\cdot \tau \right)}}d\tau \end{array} \end{array}$$

where $\omega_{K}=\beta \cdot D\cdot \omega_{\text{TS}}^{2}$ and *erf*() represents the error function.

Off-rates from intermediates were determined by fitting dwell time distributions to a model that describes the escape rate with two determinant rates (Rief et al., 2000):

(9) $$\begin{array}{l} \begin{array}{l} p\left(\tau_{1+2}\right)=\frac{k_{1}\cdot k_{2}}{k_{1}-k_{2}}\cdot \left[\exp\left(-k_{2}\cdot \tau_{1+2}\right)-\exp\left(-k_{1}\cdot \tau_{1+2}\right)\right] \end{array} \end{array}$$

where, in our use case *k*_1_ and *k*_2_ represent off-rates out of two adjoining and predominant high-energy intermediate states located along a transition path and τ_1+2_ reflects the combined dwell time of these two intermediates measured as an effective overall transition path time.

All diffusion coefficients reported are represented with contour length as a reaction coordinate.

### Simulation of Transition Path Time Distributions

Simulations of transition path trajectories were performed by integrating the discrete-time Langevin equation in a 1D potential as described previously (Ramm et al., 2014). To gather statistics, transition path times were collected using a flux sampling scheme (Zhang et al., 2007; Laleman et al., 2017).

## Results

### Low-Bandwidth Stretch-Relax Experiments of an Artificial Protein Suggest Two-State Behavior

**Figure 1:**
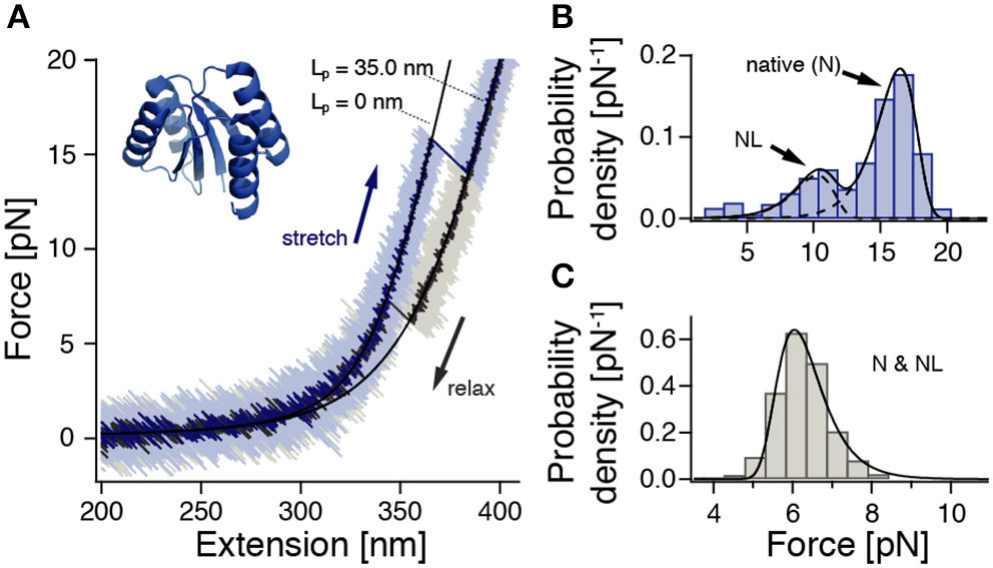
ROSS shows apparent two-state folding with minor populations of a native-like (NL) configuration. Cf. Supplementary Figure 10 for a zoom into the unfolding transition. **(A)** Stretch-relax cycle of ROSS recorded in a 500 nm/s constant-velocity experiment. Black lines are polymer model fits to the stretches of the folded and unfolded conformations. **(B)** Unfolding force distribution (500 nm/s, *n* = 691) with fits [Equation (S1)] to two distinct populations. **(C)** Refolding force distribution (500 nm/s, *n* = 651). Fit according to Equation (S2).

To study the folding and unfolding transition path times of an artificially designed globular protein, we introduced terminal cysteines into the sequence of ROSS, a 100 amino acid (aa), fully artificially designed protein that adopts a 2x2 Rossmann fold (Koga et al., 2012) (Figure 1A, inset). We then fused the protein termini to DNA handles and assembled bead-DNA-protein-DNA-bead dumbbells for force spectroscopic measurements in a custom-built optical tweezer instrument.

To characterize the mechanical behavior of ROSS, we subjected the protein-DNA dumbbell to stretch-relax cycles at 500 nm/s (Figure 1A). In the majority of cycles, ROSS displays apparent two-state unfolding behavior with unfolding forces at ≈17 pN and a contour length increase of 34.7 ± 0.9 nm, in excellent agreement with an expected length of 34.5 nm for the unfolding of 100 aa. Upon relaxation, ROSS readily refolds at a force of ≈ 6 pN back to the native contour length of 0 nm. In addition to the described behavior, we also observed, in 23% of cycles, a population of unfolding events at reduced forces of ≈ 10 pN, with a similar contour length increase of 33.8 ± 0.9 nm. An unfolding force histogram (Figure 1B) shows the occurrence of this second mechanically weaker state, which we call the native-like (NL) conformation. Refolding forces followed the expected behavior of a single pathway (Figure 1C). To obtain the equilibrium free energy difference between the native and unfolded states, we employed the Crooks fluctuation theorem (Crooks, 1999; Collin et al., 2005) (Supplementary Figure 5). We found a stability of the native state of −27 ± 2 *k*_B_*T*, in good agreement with a reported value of −25.2 *k*_B_*T* in chemical denaturation (Koga et al., 2012).

### Transition Path Times Suggest a Rough Transition Barrier

**Figure 2:**
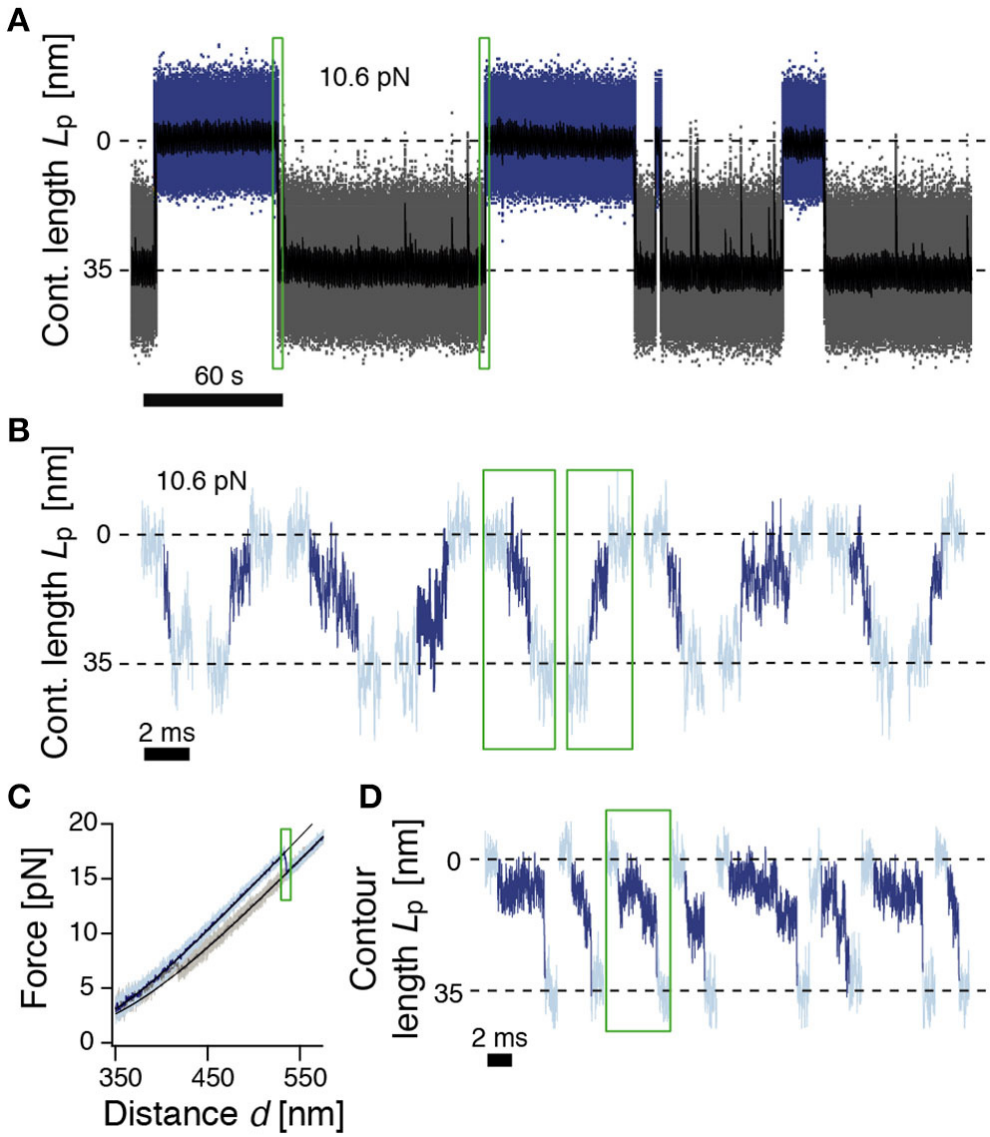
ROSS shows slow transitions between the native and unfolded states. **(A)** Passive-mode trajectory of equilibrium fluctuations between the native (blue, 0 nm) and unfolded states (gray, 35 nm of unfolded contour length). Black: Filtered trace to guide the eye. The indicated force is acting on the native state. **(B)** Extracted transitions between the native and unfolded states from passive-mode experiments. Dark colors show the identified trajectories during barrier crossing. Green boxes highlight exemplary positions in trace (A). **(C)** Force-distance curve at a pulling speed of 500 nm/s. Black solid lines are polymer model fits to the folded and unfolded conformations. **(D)** Unfolding transitions from force-distance curves. Dark colors indicate trajectories during barrier crossing. The trajectory in the green box corresponds to the trajectory shown in (C).

We sought to investigate the transition path times (TPTs) for the unfolding and folding in greater detail. To this end, we held the tethered protein under tension in passive mode and followed the folding/unfolding transitions at high bandwidth over several minutes (Figure 2A). ROSS displayed long dwells in the native (N, 0 nm contour length) and unfolded (U, 34.7 nm contour length) states of up to a minute in duration. In addition, we also observed transient short-lived excursions from U toward the native structure, which we attribute to unsuccessful folding events. Using a model that incorporates the energetic contributions of linker stretching (see **Methods**) we determined a free energy difference between the N and U states of −28 ± 3 *k*_B_*T*, confirming our earlier result from stretch-relax cycles (−27 ± 2 *k*_B_*T*) and reported values from chemical denaturation (−25.2 *k*_B_*T*) (Koga et al., 2012).

We next took a closer look into the transitions between the N and U states (Figure 2B). To identify parts of the trajectory on a barrier-crossing transition path, we used hidden Markov modeling with a transient state between N and U and considered parts of the trajectory that were classified into this transient state as barrier crossing events. We observed a diverse ensemble of trajectories, ranging from 140 μs to 5.5 ms in duration. The average folding duration (1.4 ± 0.2 ms) and unfolding duration (1.5 ± 0.2 ms) were identical, as expected (Neupane et al., 2016). Interestingly, the transitions were generally not unidirectional but showed frequent changes in direction and velocity as well as discernible pauses. Notably, the measured transition path times for ROSS were much slower than values obtained for a DNA hairpin (≈ 27 μs) (Neupane et al., 2016), the artificial protein α_3_D (≈ 15 μs) (Chung and Eaton, 2013), or dimeric prion protein PrP (≈ 500 μs) (Neupane et al., 2016). The transition path duration of ROSS was also much slower than the predicted speed limit for the folding of a protein of 100 aa (≈ 1 μs) (Kubelka et al., 2004). To validate the resulting exceptionally slow barrier crossing time, we averaged aligned folding or unfolding transitions and fitted the resulting average relaxation response to a sigmoidal function (Supplementary Figure 4). The equivalent durations from this method were (0.7 ± 0.1 ms) for folding and (1.1 ± 0.1 ms) for unfolding, close to our previous results. Interestingly, transitions into non-native conformations were much faster and averaged ≈ 0.2 ms (Supplementary Figure 4C). Taken together, our observation of an exceptionally slow folding/unfolding time hint at the presence of a rugged barrier.

We suspected that the observed barrier crossing trajectories may be convoluted by thermal fluctuations of the beads that do not directly reflect changes in the protein end-to-end distance. We reasoned that this effect may be minimized at higher forces when the tension in the tether is higher and force fluctuations are thus minimized. To this end, we deliberately drove the system out of equilibrium with constant-velocity stretch-relax cycles and recorded the relaxation of the beads back to their equilibrium position at high bandwidth (Figure 2C). We note that during folding/unfolding, the trap position movement was ≈2 nm, much smaller than the observed bead relaxation amplitudes (≈15 nm). Hence, we do not anticipate significant bias from the active pulling protocol. Figure 2D shows representative relaxation trajectories of unfolding transitions that occurred at forces higher than 15 pN, thus fully excluding transitions that originate from the NL state. Contrary to expectations from a two-state model, and confirming our results from passive mode, the beads did not rapidly settle back into their relaxed position but displayed complicated trajectories for several milliseconds until relaxation, again hinting at a rugged barrier separating states N and U.

### Transition Path Times of ROSS Depend on the Applied Force

The transition path times obtained from stretch-relax cycles probe the barrier at a different force bias and are thus not necessarily the same as those obtained from passive mode (Gladrow et al., 2019). Whereas, the N and U basins in our passive-mode experiments are approximately at the same energetic level, the U basin in typical stretch unfoldings lies ≈32 *k*_B_*T* lower than the N basin. We, therefore, investigated whether the transition path times at higher forces, where the equilibrium is shifted toward the unfolded state, differs from the situation at low forces.

In the case of a symmetric harmonic barrier of significant height, the average transition path time is $\langle \tau_{\text{TP}}\rangle \approx \frac{\ln\left(2e^{\gamma_{E}}\cdot \beta \cdot \Delta G^{‡}\right)}{\beta \cdot D\cdot \omega_{\text{TS}}^{2}}$ (Chung et al., 2009; Chaudhury and Makarov, 2010). Even though under increased force bias the approximation of a symmetric harmonic barrier no longer holds, the model predicts that 〈τ_TP_〉 is largely unaffected by a shift to higher forces due to its logarithmic dependency on the barrier height, a result that was also confirmed experimentally for a DNA hairpin (Neupane et al., 2016).

**Figure 3:**
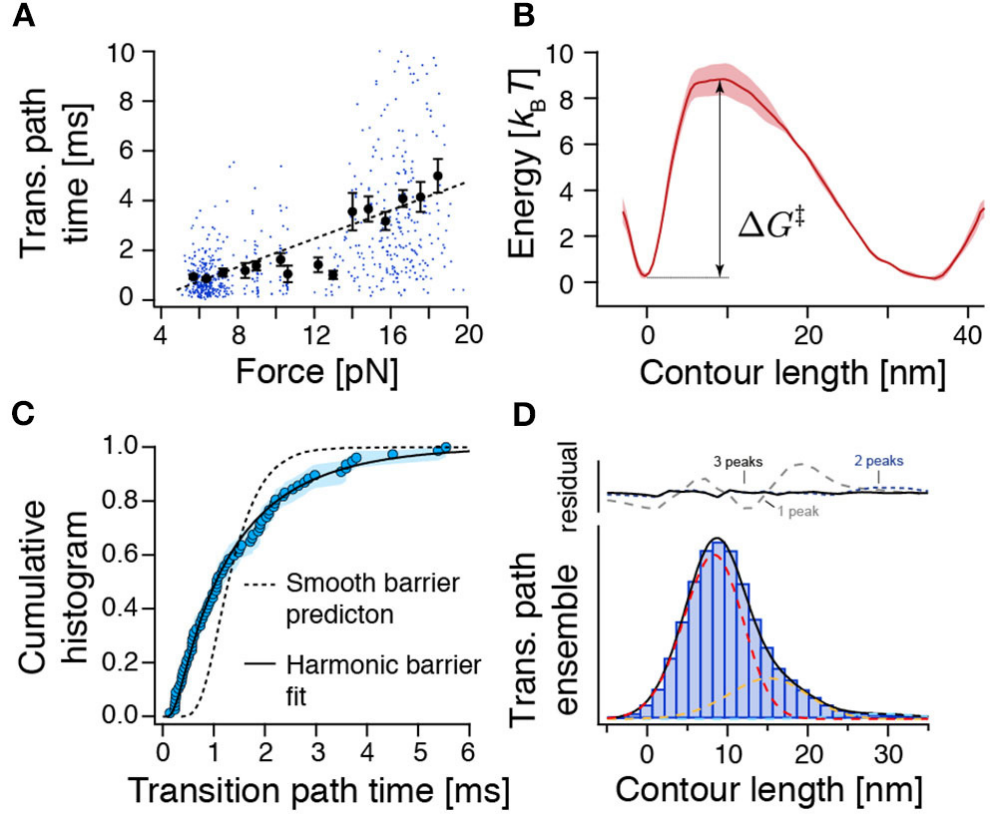
The force-dependence and distribution of transition path times as well as the transition path ensemble show unexpected results for crossing a single and smooth barrier. **(A)** Transition path time (TPT) versus force plot for measured TPTs between N and U from both constant-velocity and passive-mode experiments (*n* = 654). While dots represent individual data points, points with error bars represent average transition path times within their respective force range. The dashed line is a linear fit to guide the eye. The related Supplementary Figure 6 shows all detected transitions color coded according to their experimental origin. **(B)** Reconstructed free energy landscape from deconvolution of bead fluctuation histograms obtained from passive-mode experiments. **(C)** The distribution of experimental transition path times from passive-mode experiments (circles, *n* = 77) can be fitted to an approximation model for a central harmonic barrier [Equation (8), continuous line], albeit with unrealistic fit parameters (see text). Flux sampling simulations of one-dimensional diffusion over the deconvolved barrier shown in (B) predict a much narrower distribution (dashed line) that is incompatible with the experimental data (*p* = 5 × 10^−6^, KS test). **(D)** Position histogram of transition paths extracted from force-ramp experiments (bars, *n* = 85 transitions). Dashed lines represent fitted point-spread functions of three intermediates (see **Methods**). Black line: the sum of fitted point-spread functions. Upper panel: Fitting residuals scenarios of one, two, or three intermediates. Δ*G*^‡^ represents the height of the energy barrier as in Equation (7).

We collected a dataset of the transition path times of ROSS folding/unfolding from passive-mode experiments and stretch-relax cycles where transitions occurred in a range between 5 and 20 pN (Figure 3A, also see Supplementary Figure 6 for an annotated and expanded version that also contains NL transitions). Surprisingly, we observed a clear correlation of increasing τ_*TP*_ with higher force (*r* = 0.53 ± 0.06, 99 *% CI* from bootstrapping), in stark disagreement with predictions from a harmonic barrier model. N → U transitions that occurred at ≈17 pN were much slower (3.9 ± 0.2 ms) than passive-mode N → U transitions at ≈10 pN (1.5 ± 0.2 ms, see above) or the corresponding reverse reaction U → N at ≈6 pN (0.9 ± 0.1 ms) (Supplementary Figure 6). To understand the significance of this observation, we performed diffusion simulations in a 1D potential for the case of a smooth harmonic barrier and the case of a barrier harboring a weakly stable intermediate (Supplementary Figure 7). Corroborating our suspicion, 〈τ_TP_〉 was largely independent of force bias in the case of a harmonic barrier (Supplementary Figure 7B), but was force-dependent in the case of a barrier containing a weak intermediate (Supplementary Figure 7D). Together, these results confirmed our previous observation that ROSS folds over a rough barrier.

### Transition Path Times Are Incompatible With a Smooth Barrier

To get a more detailed picture of the folding barrier, we reconstructed the free energy landscape by deconvolving the bead fluctuation histograms obtained from passive-mode (constant-trap-separation) trajectories (Woodside et al., 2006; Gebhardt et al., 2010; Hinczewski et al., 2013; Ramm et al., 2014) (Figure 3B). However, because the time spent transitioning is an order of magnitude smaller than the time spent in the folded and unfolded states (≈0.01 %), the reconstructed energy landscape emphasized the N and U basins but failed to pick up any deviations from a smooth barrier profile. We found a single asymmetric barrier with $\Delta G^{‡}=8.8\pm 0.8\;k_{\text{B}}T$. Our measured barrier crossing time allowed us to estimate the diffusion constant of the reaction using (Hummer, 2004)

(10) $$\begin{array}{l} \begin{array}{l} D=\frac{1}{\langle \tau_{\text{TP}}\rangle }\int_{x_{A}}^{x_{B}}e^{-\beta G\left(x\right)}\Phi_{A}\left(x\right)\Phi_{B}\left(x\right)dx\int_{x_{A}}^{x_{B}}e^{\beta G\left(x\right)}dx, \end{array} \end{array}$$

where Φ_*A*_ and Φ_*B*_ are committor functions (Hummer, 2004). We found *D* = 10^4.6±0.1^nm^2^/*s*. A comparable value (*D* = 10^4.3±0.1^nm^2^/*s*) was obtained when we used the approximation of a central harmonic barrier $D\approx \frac{\ln\left(2e^{\gamma_{E}}\cdot \beta \cdot \Delta G^{‡}\right)}{\beta \cdot \langle \tau_{\text{TP}}\rangle \cdot \omega^{2}}$ (Chung et al., 2009; Chaudhury and Makarov, 2010). An estimation of the diffusion constant based on the barrier height and the measured equilibrium transition rate constants between N and U using Kramers' theory (Kramers, 1940) ($D\approx 2\pi k\frac{e^{\Delta G^{‡}}}{\beta \omega^{2}}$) yielded a similar value of *D* = 10^4.6±0.4^ nm^2^/*s*. Notably, this experimental diffusion constant is several orders of magnitude lower than the value expected for a protein that folds at its speed limit (τ_TP_ ≈ 1 μ*s*, *D* ≈ 10^7.7^ nm^2^/*s*) (Kubelka et al., 2004). Slowed diffusion can be interpreted in terms of a rough energy landscape (Zwanzig, 1988) $D=D_{0}e^{-{\left(\frac{\varepsilon_{\text{RMS}}}{k_{\text{B}}T}\right)}^{2}}$, where ε_RMS_ is a measure of the roughness of the energy profile. Based on our measurements, we estimate ε_RMS_ ≈ 2.7 *k*_B_*T*, which amounts to a sizeable ≈10% of the folding free energy.

The previous analysis only considered the average transition path time 〈τ_TP_〉 and predicted a slow diffusion coefficient. We next also tested if the distribution of experimental transition path times is compatible with a one-dimensional diffusion model for the deconvolved barrier profile (Figure 3C). However, we found that the model (flux sampling calculation with *D* = 10^4.6^nm^2^/*s*, dashed line) predicts a much narrower distribution than the experimental data. An approximation model for a central harmonic barrier [Equation (8), continuous line] could be fitted to the data. Nevertheless, the obtained parameters of *D* ≈ 10^3.8^ nm^2^/*s* and $\Delta G^{‡}\approx 0.2\;k_{\text{B}}T$ were incompatible with the smooth-barrier estimations obtained earlier (*D* = 10^4.6±0.1^nm^2^/*s*, $\Delta G^{‡}=8.8\pm 0.8\;k_{\text{B}}T$). Notably, a similar discrepancy has been observed also for PrP (Neupane et al., 2016). We conclude that the smooth barrier obtained from deconvolution (Figure 3B) is incompatible with the wide distribution of observed transition path times.

### Weak Transient Intermediates Cause Slow Transition Paths

To understand the extraordinarily slow TPTs of ROSS, we took a closer look at the ensemble of trajectories during barrier crossing *p*(*x*|TP). Figure 3D shows a position histogram of transition paths obtained from force-ramp experiments as shown in Figure 2D. Interestingly, the measured histogram was peaked at ≈9 nm, which is unexpected for a symmetric barrier (Hummer, 2004) but is in good agreement with the location of the identified barrier position from deconvolution (Figure 3B). The same indication of an asymmetric barrier was found in passive-mode experiments (Supplementary Figure 8).

We suspected that the protein populates weakly metastable on-pathway intermediates that slow down the overall transition and thus give rise to the notion of a rough energy landscape. We reasoned that the population of intermediates may produce elongated pauses at certain positions along the reaction coordinate of unfolded contour length that should be visible in transition path ensembles. Indeed, when comparing *p*(*x*|*TP*) with the point-spread function (PSF) for intermediates, we noticed that at least three intermediates are necessary to fit the experimental *p*(*x*|TP) (Figure 3D). While it is technically not possible to assign thermodynamically correct population densities to the intermediates, because of the non-equilibrium way the transition paths to produce *p*(*x*|TP) are selected, the described procedure nevertheless clearly hints at the population of intermediates.

While intermediates were hidden in deconvolution of the equilibrium distribution (Figure 3B), the intermediates can be readily seen when the deconvolution procedure was applied to the transition path ensemble *p*(*x*|TP) (Supplementary Figure 9), where the selection emphasizes parts of the trajectory that cross the barrier. However, because of this selection, the obtained probability distribution cannot be Boltzmann inverted to obtain a reconstruction of the barrier.

### Reconstruction of the Energy Barrier

**Figure 4:**
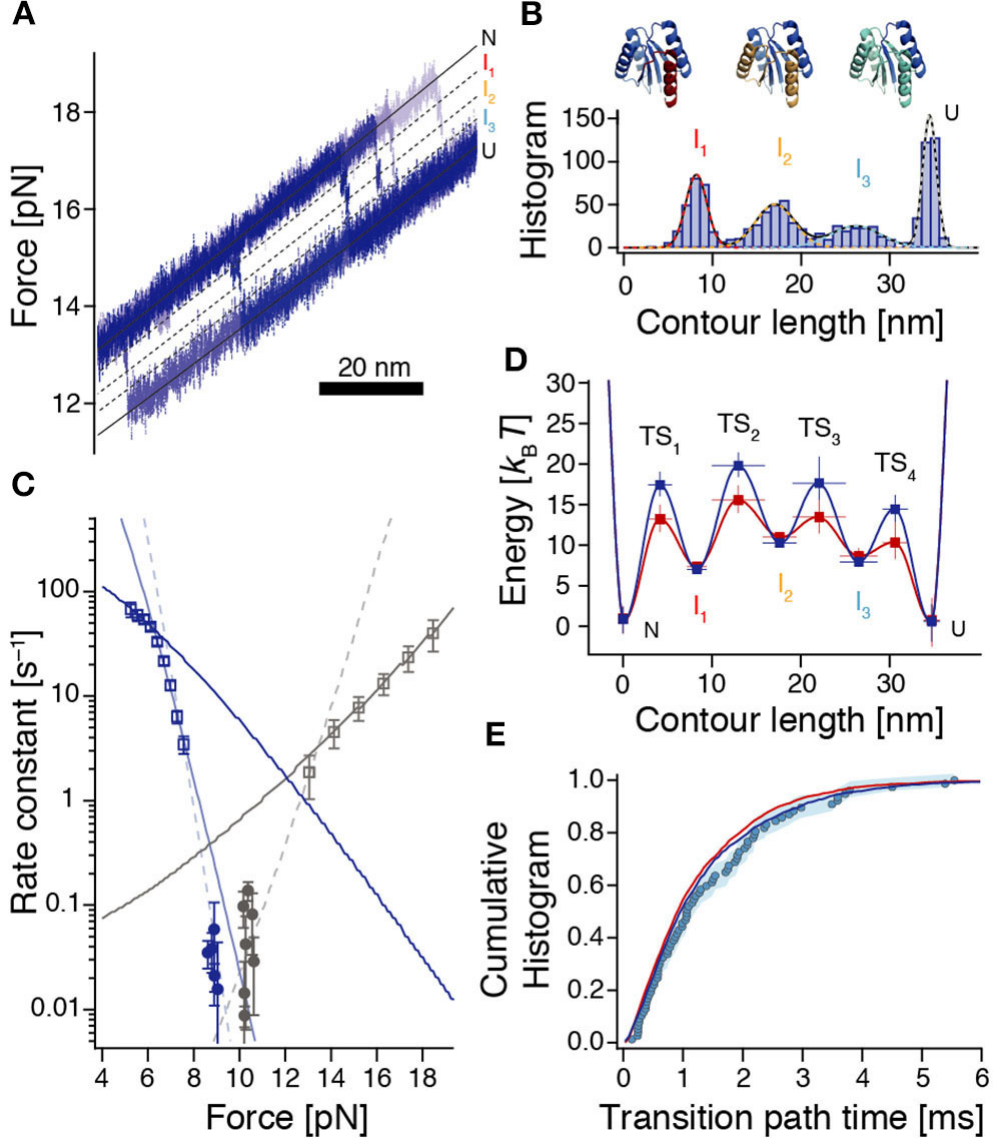
Reconstruction of the rough energy landscape of ROSS. **(A)** Example of six high-resolution unfolding trajectories from constant-velocity experiments used to reveal positions of high-energy intermediates. Black lines indicate the corresponding positions. **(B)** Contour length gain histogram based on WLC-fits to unfolding events from force-ramp experiments (*n* = 1010 from 304 unfolding events). Colored dashed lines are fits to the individual peaks resulting from a Gaussian multi-peak fit to the entire histogram. Top: Assignment of unfolded structural elements of ROSS to the intermediates. **(C)** Force-dependent rates for folding (dark blue) and unfolding (gray) of ROSS extracted from constant-velocity (squares) and passive-mode experiments (circles). Continuous lines are local fits [Equation (S6)] to the data. Dashed lines interpolate between the accessible force ranges of constant-velocity and passive-mode experiments and illustrate the notion of switches of the dominant transition barrier in different force regimes. **(D)** Reconstructed energy landscape for ROSS. For the dark blue landscape, we assumed that folding transitions are fundamentally limited by speed limit protein folding (*D* ≈ 10^7.7^ nm^2^/*s*); for the dark red landscape, we assumed that the speed of our observation is fundamentally limited by the bead diffusion in our optical trap (*D* ≈ 10^6^ nm^2^/*s*). See Supplementary Tables 1, 2 for parameters of the energy landscapes. **(E)** The experimentally derived transition path time distribution (circles, same data as in Figure 3C) is well-reproduced by the distributions predicted from the energy landscapes shown in (D) [dark blue (*p* = 0.22, KS test) and red (*p* = 0.12, KS test) lines, respectively].

We, therefore, reverted to approximating the energy barrier based on force-dependent transition rate constants between N and U. WLC-model fits to high-force unfolding trajectories revealed the positions of three high-energy intermediates I_1_-I_3_ (Figures 4A,B, Supplementary Figure 10). We obtained *L*_*p*_(I_1_) = 8.3 ± 1.1 nm, *L*_*p*_(I_2_) = 17.6 ± 1.9 nm, *L*_*p*_(I_3_) = 26.5 ± 2.1 nm, where *L*_*p*_(N) = 0 nm.

To identify the position of the dominant transition barrier we determined the force-dependent rate constants for folding and unfolding (Figure 4C), where we used both data from passive-mode and stretch-relax experiments, and fitted a model that incorporates the energetic contributions of all compliances in the system (Schlierf et al., 2007). Notably, this model assumes that transition state positions are independent of the applied force. Therefore, kinks in the force-dependent rate plots or corresponding deviations from the model indicate a switch of the dominant transition barrier (Schlierf et al., 2010; Rico et al., 2019). Based on unfolding and refolding events observed during constant-velocity measurements, we identified three force ranges where extracted rates could be fitted locally (Figure 4C, continuous lines). In addition, rate constants extracted from passive-mode experiments suggest that there must be at least one more force range with a different predominant transition state since these rates are substantially lower than any of the three fits would suggest. Taken together, this yields four required transition states to interpret our data, in agreement with our finding of three intermediate states I_1_, I_2_, and I_3_ between N and U. The force at which the TS_3_:TS_4_ transition state switch occurs, lies at around 6.3 pN (force of U), which is directly given by the intersection of the respective rate fits. The transition state switches TS_2_:TS_3_ and TS_1_:TS_2_ occur around 7.5 pN and 13 pN, respectively. The slopes from the fitted rate constants indicate transition state positions TS_1_ = 3.1 ± 0.3 nm between N and I_1_, TS_3_ = 19.4 ± 1.1 nm between I_2_ and I_3_, as well as TS_4_ = 30.5 ± 1.3 nm between I_3_ and U. Due to the very slow folding kinetics and the narrow accessible force range for measurements in passive-mode, no position could directly be derived from our data for TS_2_. However, interpolations between rate constants obtained from stretch-relax cycles and passive-mode experiments suggest that the TS_2_ position is situated at ≈ 10 – 12 nm which is right between I_1_ and I_2_ (Figure 4C, dashed fits).

To obtain values for the energies of the intermediates and transition states, we employed a piecewise reconstruction method where we determined individual effective barrier heights for each dominant barrier position [i.e., at different force biases, based on the measured dwell time distribution and Kramers' rate equation (Hänggi et al., 1990, Supporting Methods)]. Reasoning that the experimentally determined transition path times are likely dominated by metastable dwells in high-energy intermediates, we fitted the transition path time distribution to a model where the barrier crossing time is determined by the sequential population of the three intermediates I_1_, I_2_, and I_3_. The corresponding rate constants for the escape rates from I_1_, I_2_, and I_3_ were then translated into barrier heights using Kramers' rate equation, where we assigned the longest dwell to I_1_ and the shortest dwell to I_3_, in line with our observations (Figures 2B,D, 3D). Finally, we used the experimentally determined rate constants between N and U and the switch of the dominant transition barrier at different force bias to determine the missing barrier heights (Supplementary Figure 11 and Supporting Methods).

All reconstruction steps depend on an assumption of the diffusion constant *D*. Since bumps in the energy profile slow down the observed transitions, our experiments imply that a realistic intrinsic *D* must be higher than the smooth barrier estimation of *D* ≈ 10^4.6^ nm^2^/*s*. Here we did the reconstruction for two cases: The assumption that folding is fundamentally limited by the folding speed limit (*D* ≈ 10^7.7^ nm^2^/*s*) and the assumption that the speed of our observations is fundamentally limited by the diffusion of the beads in the optical trap (*D* ≈ 10^6^ nm^2^/*s*). The resulting barrier reconstructions mostly only differ in the barrier heights and are shown in Figure 4D.

### A Barrier Harboring High-Energy Intermediates Agrees With Pulling Variants and Reproduces Experimental Transition Path Time Distributions

To verify our barrier reconstruction, we again used flux sampling simulations to determine the distribution of transition path times, based on the reconstructions shown in Figure 4D. In contrast to the smooth barrier case, this barrier profile yielded a much wider transition path time distribution that matched the experimental data very well (Figure 4E). In addition, we also performed Langevin dynamics simulations within the energy landscapes of Figure 4D to show that the ensembles of transition paths predicted from the reconstructions agree with experimental distributions (Supplementary Figure 12).

Corroborating evidence for the validity of our energy landscape reconstruction comes from a directional pulling mutant of ROSS, S49Cc, where force was only applied between residue 49 and the c-terminus. This variant also harbored an intermediate, which matched the I_1_ intermediate of ROSS both in length (8.0 ± 0.9 nm) and in energy (−15 ± 2 *k*_B_*T*) (ROSS: 8.3 ± 1.1 nm and −15 ± 3 *k*_B_*T*, respectively; see Supplementary Figures 13, 14). In addition, the obligatory intermediate of S49Cc attains its maximal population at ≈13 pN, with an average dwell time of ≈2 ms. This closely resembles the properties of the corresponding I_1_ intermediate of ROSS, which also reaches its maximum population at ≈13 pN, with a comparable lifetime (Supplementary Figure 15). Taken together, these results suggest that typical ROSS unfolding occurs via the unfolding of the c-terminal half of the protein, where the length of the first intermediate is compatible with the unraveling of the c-terminal helix.

As shown earlier, metastable intermediates in the barrier may cause a shift in the average transition path time with varying force (cf. Supplementary Figure 7). We determined the predicted variation of 〈τ_TP_〉, based on the energy landscapes of Figure 4D analytically and with simulations. While both energy profiles correctly predicted an increase of 〈τ_TP_〉 with increasing force, both also predict a subsequent decrease above about 13 pN (Supplementary Figure 15). While this behavior was directly observed in the variant S49Cc (Supplementary Figure 14C), the same trend reversal was not apparent in experimental data of ROSS (Figure 3A), suggesting that perhaps, a one-dimensional description of the energy barrier may not be adequate for ROSS, or the non-equilibrium protocol used for obtaining transition path times at high forces may probe different transition paths (Gladrow et al., 2019).

## Discussion

In summary, ROSS exhibits remarkably slow transitions over the barrier between the folded and unfolded states, much slower than the transitions of other systems observed in optical tweezers (Neupane et al., 2016; Hoffer et al., 2019). FRET experiments generally have yielded even faster transition path times (Chung et al., 2012; Chung and Eaton, 2013). However, timescales are not directly comparable between different experimental techniques, because of the time-limiting effect of the measurement apparatus (Cossio et al., 2015, 2018; De Sancho et al., 2018). The best-characterized system in optical tweezers, DNA hairpins, generally displays much faster transitions than proteins (Neupane et al., 2016), likely because their transitions, owing to the experimental unzipping geometry, are well-described by a one-dimensional diffusion model (Neupane et al., 2012).

Slow transition path times have been discussed in the context of internal friction, frustration, or energy profile roughness (Liu et al., 2009; Wensley et al., 2010; Borgia et al., 2012; Chung et al., 2015), but our analysis revealed that none of these can explain the wide transition path time distribution of ROSS. Instead, we found evidence of a series of on-pathway intermediates that are briefly populated during barrier crossing. A similar mechanism has been observed for the coupled folding and binding of IDPs (Sturzenegger et al., 2018).

The slow transition path times of ROSS raise the interesting possibility that its impeded folding may be a consequence of its origin in artificial design. Indeed, most design algorithms, such as Rosetta optimize an equilibrium low energy state of the native target structure, compared to non-native interactions (Koga et al., 2012), but not the pathway of folding. The high-energy intermediates observed in our measurements may be caused by improper packing of individual alpha or beta secondary structures. The population of these non-native contacts, especially under denaturing conditions, may effectively hinder efficient folding. On the other hand, high-energy intermediates may also act as “checkpoints” in the folding landscape that can aid folding along a specific pathway, at the expense of overall cooperativity.

Notably, Rosetta structure prediction simulations indicate that ROSS may indeed adopt a non-native fold that is energetically very close to the native design structure (Koga et al., 2012). It is unclear if we observe this conformation in our experiments. However, possible candidates are the NL states and fast probing events observed in both variants. Interestingly, the fraction of NL states in the pulling variant S49Cc, where force is only applied across the c-terminal half, is significantly reduced from ≈23 to ≈7% (*p* < 0.01, binomial test), suggesting that NL states may be caused by non-native interactions between the n-terminal and the c-terminal halves.

Interestingly, the extremely slow transition path time of ROSS was only apparent in transitions between the native and unfolded conformations. Other transitions, such as incomplete folding events (Supplementary Figure 4C), were faster by almost an order of magnitude. In the context of a one-dimensional energy landscape, these probing events may correspond to partial barrier crossings from U to I_1_. Indeed, our model predicts that such a partial crossing of the barrier lasts ≈150 μs, close to experimental values. However, this scenario fails to explain why under passive-mode conditions, there appear to be more probing events than successful folding transitions, requiring that the barrier TS_1_ must be higher than TS_2_. Therefore, an alternative explanation may be that the energy landscape of ROSS is multi-dimensional and our reconstruction only captures a projection of the true energy landscape.

A scenario of multidimensionality may result in position-dependent diffusion along the projected experimentally accessible energy landscape and, under certain circumstances, also result in a distribution of TPTs that is compatible with experiments. Supplementary Figure 16 illustrates this at the example of the smooth barrier of Figure 3B, where the diffusion coefficient was set to *D* = 10^7.7^ nm^2^/s everywhere except in a close neighborhood around I_1_ where we set *D* = 10^3.4^ nm^2^/s. However, while this model can also reproduce the experimental TPT distribution (Supplementary Figure 16B), it fails to explain the experimentally observed force dependence of 〈τ_TP_〉 (Supplementary Figure 16C). While it may be possible to build a position-dependent diffusivity model that also reproduces a force-dependent 〈τ_TP_〉 (e.g., by requiring a force-dependent change of *D*), the basis for this is rather speculative. On the contrary, the reconstructions of Figure 4D reproduce both the distribution and force-dependence of TPTs, without the need for additional assumptions.

Finally, our results allow us to infer a structural interpretation of the rearrangements during barrier crossing (Figure 4B, top). Unfolding of ROSS starts via I_1_ which can be attributed to the unfolding of the c-terminal α-helix., while the proximate β-sheet remains bound to the hydrophobic core. After I_1_, unfolding continues via I_2_ which can be attributed to the unfolding of the first three structural elements α*βα* starting from the c-terminus. The last and very weak intermediate I_3_ most likely represents a conformation where only the n-terminal β-sheet and the successive α-helix remain folded, a possible folding seed for ROSS.

In summary, here we have presented a single-molecule folding study of the artificially designed protein ROSS. The protein showed exceptionally slow transition path times that are incompatible with a smooth transition barrier but could be explained by the presence of high-energy intermediates. Our results illustrate that high-energy folding intermediates slow the barrier crossing of all reaction systems and raise the question of whether uncharacteristically slow transition path times may be a trait of many protein structures that have been optimized for folding stability instead of folding speed (Basak et al., 2019).

## Data Availability Statement

The raw data supporting the conclusions of this article will be made available by the authors, without undue reservation.

## Author Contributions

AM conducted experiments. JS and AM analyzed data. BP, JF, and HL contributed reagents and methods. JS conceived of the study and wrote the paper with input from all authors. All authors contributed to the article and approved the submitted version.

## Conflict of Interest

The authors declare that the research was conducted in the absence of any commercial or financial relationships that could be construed as a potential conflict of interest.
