# Supplementary material for: Slow Transition Path Times Reveal a Complex Folding Barrier in a Designed Protein
Source: Front Chem. 2020 Dec 7;8:587824. doi: 10.3389/fchem.2020.587824 (PMC7750197; doi:10.3389/fchem.2020.587824)
Supplement: Supplementary file 1 [file Data_Sheet_1.pdf]

Supporting information for:

Slow transition path times reveal a complex folding barrier in a designed protein

Alexander Mehlich[1], Jie Fang[2], Benjamin Pelz[1], Hongbin Li[2], Johannes Stigler[3,*]

[1] Physics Department E22, Technische Universität München, Garching, Germany

[2] Department of Chemistry, University of British Columbia, Vancouver, Canada

[3] Gene Center Munich, Ludwig-Maximilians-Universität München, Munich, Germany

[*] To whom correspondence should be addressed: stigler@genzentrum.lmu.de

Supporting methods

Unfolding and refolding force distributions

To fit unfolding and refolding force distributions we solved $p(F) = \frac{k(F)}{|\dot{F}|} \cdot \exp\left(-\int_{F_{\text{start}}}^{F} \frac{k(F')}{\dot{F}'} \mathrm{d}F'\right)$ with $k(F) = k^0 \cdot \exp\left(\frac{F \cdot \Delta x}{k_{\text{B}}T}\right)$. Here, F represents the acting force, $\dot{F} = \kappa_{\text{eff}} \cdot v$ is the loading rate given be the effective spring constant κ_{eff} times pulling or relaxation velocity v. Further, Δx represents the transition state position and k^0 is the zero-force rate constant.

For unfolding force distributions, we integrate from F_{start} equal to zero up to F and obtain

$$p_{\text{unf}}(F) = \frac{k_{\text{unf}}^0}{\kappa_{\text{eff}} \cdot v} \cdot \exp\left\{\frac{F \cdot \Delta x}{k_{\text{B}}T} - \frac{k_{\text{unf}}^0 \cdot k_{\text{B}}T}{\kappa_{\text{eff}} \cdot v \cdot \Delta x} \cdot \left[\exp\left(\frac{F \cdot \Delta x}{k_{\text{B}}T}\right) - 1\right]\right\} \tag{S1}$$

For folding, we obtain

$$p_{\text{fold}}(F) = -\frac{k_{\text{fold}}^0}{\kappa_{\text{eff}} \cdot v} \cdot \exp\left\{\frac{F \cdot \Delta x}{k_{\text{B}}T} - \frac{k_{\text{fold}}^0 \cdot k_{\text{B}}T}{\kappa_{\text{eff}} \cdot v \cdot \Delta x} \cdot \exp\left(\frac{F \cdot \Delta x}{k_{\text{B}}T}\right)\right\} \tag{S2}$$

The formula for $p_{\text{fold}}(F)$ is very similar to $p_{\text{unf}}(F)$. However, for folding force distributions, both the velocity v and the transition state distance Δx have negative values. In addition, we made the simplifying assumption that relaxation cycles always start at a high enough force where refolding is impossible which sets the probability to zero.

Free energy calculation

When a protein is in state i at force F_i, the Gibbs free energy of the entire dumbbell system is given by:

$$G_i(F_i) = G_i^0 + G_i^{\text{sys}}(F_i) = G_i^0 + G^{\text{bead}}(F_i) + G^{\text{DNA}}(F_i) + G_i^{\text{prot}}(F_i) \tag{S3}$$

where G_i^0 denotes the free energy of the protein in state i and $G_i^{\text{sys}}(F_i) = G^{\text{bead}}(F_i) + G^{\text{DNA}}(F_i) + G_i^{\text{prot}}(F_i)$ represents the mechanical energy of the Hookean bead deflection G^{bead}, the stretching of the eWLC DNA handles G^{DNA} and the stretching of the WLC unfolded polypeptide chain G_i^{prot}. The non-harmonic contributions G^{DNA} and G_i^{prot} are obtained from integrals over the respective polymer models **eqns. (1),(2)**.

When a protein undergoes a conformational change from state i to state j, the accompanying contour length change causes a change in acting force from F_i to F_j with the resulting free energy difference being given by:

$$\Delta G_{ij}(F_i, F_j) = G_j(F_j) - G_i(F_i) = \Delta G_{ij}^0 + \Delta G_{ij}^{\text{sys}}(F_i, F_j) \tag{S4}$$

A way to assess folding free energy differences from experimental data, is by measuring the force-dependent state occupancy $P_i(F_i)$ for being in state i and fitting this globally to an equilibrium model ($\beta^{-1} = k_{\text{B}}T$):

$$P_i(F_i) = \frac{1}{1 + \sum_{j \neq i} \exp\left(-\beta\left(\Delta G_{ij}^0 + \Delta G_{ij}^{\text{sys}}(F_i, F_j)\right)\right)} \tag{S5}$$

Since $\Delta G_{ij}^{\text{sys}}(F_i, F_j)$ can be calculated based on experimentally accessible data, ΔG_{ij}^0 remains as a fit parameter.

Extrapolation model for rate constants

The extrapolation model for the force-dependent rate constants takes into account the non-harmonic contributions of the DNA and polypeptide linkers and has been introduced previously (3, 4). In brief, the model is parameterized by k_0, the zero-force rate constant for a transition, and $\Delta L_{i\ddagger}$, the contour length change associated with a transition from state i to the transition state ‡, and is given by

$$k(F_i) = k_0 \exp\left(-\beta \Delta G_{i\ddagger}^{\text{sys}}(F_i, F_\ddagger)\right), \tag{S6}$$

where $\Delta G_{i\ddagger}^{\text{sys}}(F_i, F_\ddagger) = \Delta G^{\text{bead}}(F_i, F_\ddagger) + \Delta G^{\text{DNA}}(F_i, F_\ddagger) + \Delta G^{\text{prot}}(F_i, F_\ddagger)$.

Barrier reconstruction

To calculate expected barrier heights, we used Kramers' rate equation in the Smoluchowski limit (see main text **Methods**). For transition state positions we chose to use the middle between adjoining states which is supported by the positions derived from the rate plots and which allows a more general and simple approach for our reconstruction.

The main steps along our piecewise barrier reconstruction are as follows. Based on temporal and spatial information that we retrieved from transition path times and transition path ensembles, we reconstructed three barrier heights which govern the overall measured transition path times required for total barrier crossing in our passive mode experiments. Namely, these barriers are ΔG_{I1TS1}, ΔG_{I2TS3}, and ΔG_{I3TS4} (**Fig. S11A**). We then tilted these barrier heights to forces where a transition state switch is observed and were able to derive the height of the missing adjoining barrier based on the fact that the escape rate out of the intermediate between the two transition states involved in the switch must be equal over both transition states. This way we derived ΔG_{I1TS2}, ΔG_{I2TS2}, and ΔG_{I3TS3} (**Fig. S11B,C,D**). Finally, we made use of the measured overall transition rates at very high and very low forces, where the outermost transition states TS_1 and TS_4 dominate the overall unfolding and folding reaction. This allowed reconstruction of ΔG_{NTS1} and ΔG_{UTS4} (**Fig. S11B,D**).

To get into more detail, **Fig. S11** shows schematic illustrations of all energy landscape reconstruction scenarios which use temporal and spatial information extracted from experimental transition path times, transition path ensembles, and effective rate constants between the folded (N) and unfolded (U) state of ROSS. **Fig. S11A** depicts the energy landscape scenario during equilibrium fluctuations of ROSS in passive mode where transition state TS_2 dominates, as suggested by measured rates and their resulting chevrons with multiple kinks (**Fig. 4C**). Within all passive-mode experiments, the average force acting on the folded state

was 10.2 pN. In this scenario, measured transition path times required to cross the barrier between N and U reflect the sum of all dwell times needed to pass through I_1, I_2, and I_3 rather than the time needed to cross the individual barriers separating all five states. Note that due to the given scenario where all transition states are at different energy levels, only one single barrier dominates the dwell times within I_1, I_2, and I_3, i.e., the lower one. Dwell times τ_1^{DW} = 1.25±0.2 ms and τ_2^{DW} = 190±60 µs were derived from fitting the passive mode transition path time distribution shown in **Fig. 3C** to **eq. (9)**. Even though I_3 could not be clearly resolved within passive mode experiments, we could estimate a dwell time of τ_3^{DW} = 75±25 µs spent within I_3 based on its relative contribution to the overall transition path ensemble (**Fig. 3D**). Given these three dwell times, the red energy barriers marked in **Fig. S11A** were readily calculated by numerically solving Kramers' rate equation for ΔG with a given Δx and a known transition rate over ΔG given by the inverse of our dwell times. In a next step, we exploited the special condition of three transition state switches that we observed at different forces as illustrated in **Fig. S11B,C,D**. In each of our transition state switch scenarios, two neighboring transition states equally control overall folding and unfolding transitions. Hence, when residing in the intermediate i between transition states TS_i and TS_{i+1}, the rate $k_{i(i-1)}$ over TS_i must be equal to the rate $k_{i(i+1)}$ over TS_{i+1}. Therefore, if one of the two barrier heights and its inferred transition rate is known, the other barrier can be calculated by setting $k_{i(i-1)} = k_{i(i+1)}$ and again solving Kramers' rate equation with the respective Δx. To this end, we tilted each known barrier from the initial reconstruction step towards the force where the transition state switch occurred that barrier was involved in. Based on the height of the known and tilted barrier, we derived the respective adjoining barrier, which, under perfect symmetric conditions, would be directly given by $\Delta G^{i\mathrm{TS}_i} = \Delta G^{i\mathrm{TS}_{i+1}}$. Finally, we made use of another condition at very high and very low forces where the outermost barriers determine our measured effective rate constants as illustrated in **Fig. S11B,D**. In principle, this corresponds to the 'standard' use case of applying Kramers' rate equation where a measured rate is controlled by a single barrier.

Finally, all calculated barrier heights are tilted towards the scenario in **Fig. S11A** where a force of 10.2 pN acts on state N and maxima and minima of the barriers are aligned to seamlessly pass into one another. For the reconstructed energy landscapes shown in **Fig. 4D**, we included the equilibrium condition of states N and U being on approximately the same energetic level as observed in our passive-mode experiments.

Errors for barrier heights indicated in **Fig. 4D** were estimated from varying transition state distances in our calculations within the indicated error range of the respective transition state. In addition, we added an error by varying rates/dwell times by a factor of 2 within our calculations. Errors for our chosen central transition state positions were chosen such that their corresponding transition state positions inferred by our measurements would at least be included within error. Positional errors for states I_1, I_2, I_3, and U were taken from contour length gain histograms based on WLC-fits.

At zero force, the barrier reconstruction yields the following free energy differences from the state N: $\Delta G_0^{\mathrm{I}_1}$ = 15±3 $k_{\mathrm{B}}T$, $\Delta G_0^{\mathrm{I}_2}$ = 26±3 $k_{\mathrm{B}}T$, $\Delta G_0^{\mathrm{I}_3}$ = 31±3 $k_{\mathrm{B}}T$, and ΔG_0^{U} = 30±5 $k_{\mathrm{B}}T$. We note that the last value represents the folding free energy of the full protein that has been independently obtained in constant-velocity (27±2

k_BT) and passive-mode experiments (28±3 k_BT). As expected, the values agree, validating the reconstruction. All reconstructed barriers and derived free energy differences are listed in **Tables S1,S2**.

References

1. Evans E, Ritchie K (1997) Dynamic strength of molecular adhesion bonds. *Biophys J* 72(4):1541–1555.

2. Bell GI (1978) Models for the specific adhesion of cells to cells. *Science* 200(4342):618–627.

3. Schlierf M, Berkemeier F, Rief M (2007) Direct observation of active protein folding using lock-in force spectroscopy. *Biophys J* 93(11):3989–3998.

4. Gebhardt JCM, Bornschlögl T, Rief M (2010) Full distance-resolved folding energy landscape of one single protein molecule. *PNAS* 107(5):2013–2018.

5. Azzalini A (1985) A class of distributions which includes the normal ones. *Scandinavian journal of statistics* 12(2):171–178.

Supporting Tables

Barrier	N·TS_1	TS_1·I_1	I_1·TS_2	TS_2·I_2	I_2·TS_3	TS_3·I_3	I_3·TS_4	TS_4·U
$\Delta x^{i\mathrm{TS}}$ [nm]	4.15±1.35	4.15±1.35	4.65±3	4.65±3	4.45±3	4.45±3	4.1±1.4	4.1±1.4
$\Delta G^{\ddagger}$(speed) [$k_{\mathrm{B}}T$]	17.1±3.4	-10.1±1.9	13.3±1.9	-9.1±2.1	7.8±3.7	-9.4±3.9	6.9±2.4	-13.7±4.2
$\Delta G^{\ddagger}$(bead) [$k_{\mathrm{B}}T$]	12.9±3.6	-5.5±2.1	8.7±2.1	-4.2±2.2	2.9±4.3	-4.4±4.8	2±3.7	-9.4±5.9

Table S1. Reconstructed barriers for folding transitions of ROSS under a force of 10.2 pN being applied to the folded state N. Here, diffusion coefficients of $D \approx 10^{7.7}\ \mathrm{nm}^2/\mathrm{s}$ and $D \approx 10^{6}\ \mathrm{nm}^2/\mathrm{s}$ were used for barrier reconstruction of $\Delta G^{\ddagger}$(speed) and $\Delta G^{\ddagger}$(bead). The indicated barrier positions and heights result from going through the reconstructed energy landscapes depicted and correspondingly labeled in **Fig. 4D** from left to right.

	Folded native (N)	Intermediate #1 (I_1)	Intermediate #2 (I_2)	Intermediate #3 (I_3)	Unfolded (U)
L_p [nm]	0	8.3±1.1	17.6±1.9	26.5±2.1	34.7±0.9
ΔG_0(speed) [$k_{\mathrm{B}}T$]	0	15±3	26±3	31±3	30±5
ΔG_0(bead) [$k_B T$]	0	15±3	27±3	32±3	31±6

Table S2. Summary of key parameters characterizing the reconstructed energy landscape with three intermediates tilted to zero force. The value L_p represents the unfolded contour length and ΔG_0 the zero force folding free energy difference with respect to the native state (N). For ΔG_0(speed) and ΔG_0(bead), diffusion coefficients of $D \approx 10^{7.7}\ \mathrm{nm}^2/\mathrm{s}$ and $D \approx 10^{6}\ \mathrm{nm}^2/\mathrm{s}$ were used for the reconstruction, see **Table S1**.

Supporting figures

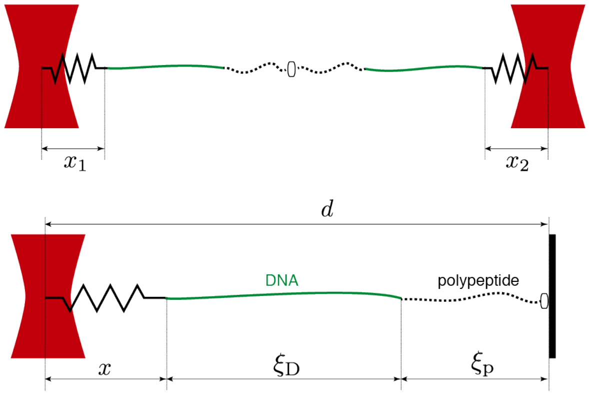

Figure S1. Schematic of the relevant lengths in our assay: Green: DNA, dashed: unfolded polypeptide. Top: Actual configuration, bottom: Equivalent configuration where the two traps are combined into one. Omitting the extension of the folded protein, the trap distance is $d = x(F) + \xi_D(F) + \xi_p(F)$, where $x = x_1 + x_2$ is the combined bead deflection. See Methods for equations describing the polymer models.

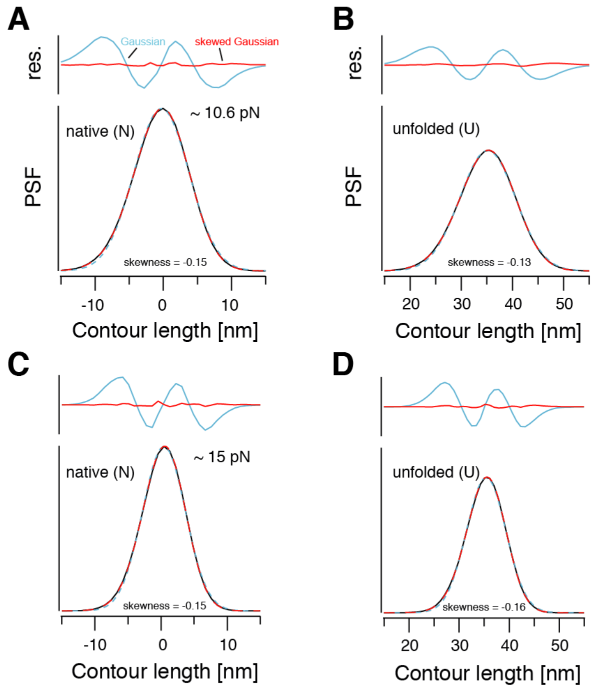

Figure S2. The point-spread functions (PSFs) of unfolded contour lengths are well-described by a skewed Gaussian. **(A)** Point-spread function calculated for a passive-mode trajectory where the native state (N) is being held at a force of about 10.6 pN (black line in the background). Dashed lines are a Gaussian (light blue) and a skewed Gaussian (red) fit to the point-spread function. Top: Fit residuals. **(B)** Point-spread function for the unfolded state (U) derived from the same equilibrium trajectory that was used in (A). **(C)** and **(D)** correspond to (A) and (B) but with a native-state force typical for stretch-relax unfolding events (15 pN).

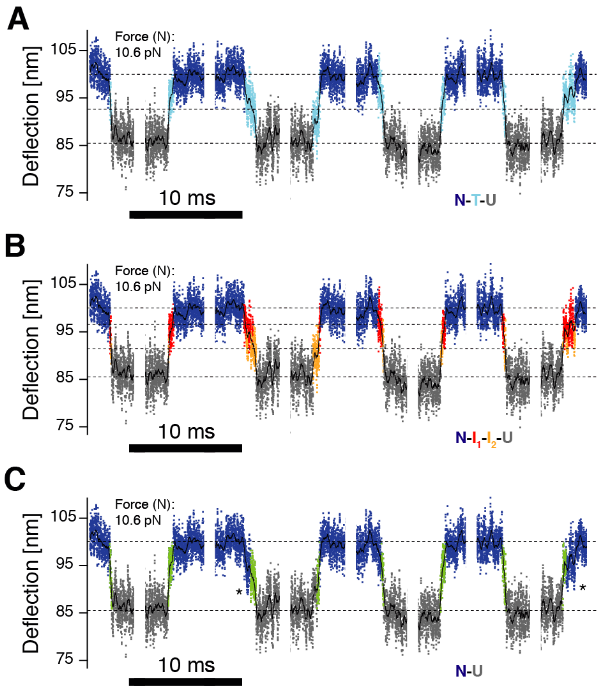

Figure S3. Comparison of the extraction of transition paths from passive-mode experiments. **(A)** One transient state T right in the middle between the fully folded N and unfolded state U. For the shown 8 transitions, the extracted average transition path time is 1.1±0.2 ms. **(B)** Two intermediates around the positions of I_1 and I_2. For the shown 8 transitions, the extracted average transition path time is 1.2±0.3 ms. **(C)** Model where re-crossings of a boundary are not allowed. However, because of thermal noise, these apparent re-crossings appear frequently and lead to an underestimation of the extracted average transition path time (0.7±0.1 ms for the shown 8 transitions). Asterisks (*) mark transitions where this model wrongly assigns stretches to the native state N (blue) that are visibly incompatible with the expected distribution of N. We note that the artificial underestimation of TPTs in this model is exacerbated at high bandwidth, when the noise of the thermal bead fluctuations is high. When resampling our 200 kHz data at 20 kHz, the resulting average transition path time is 1.2±0.2 ms, in agreement with the methods of (A) and (B) (data not shown). Taken together, this highlights the fact that high temporal resolution comes at the cost of increased thermal noise which we overcome by our HMM-based approach to determining TPTs.

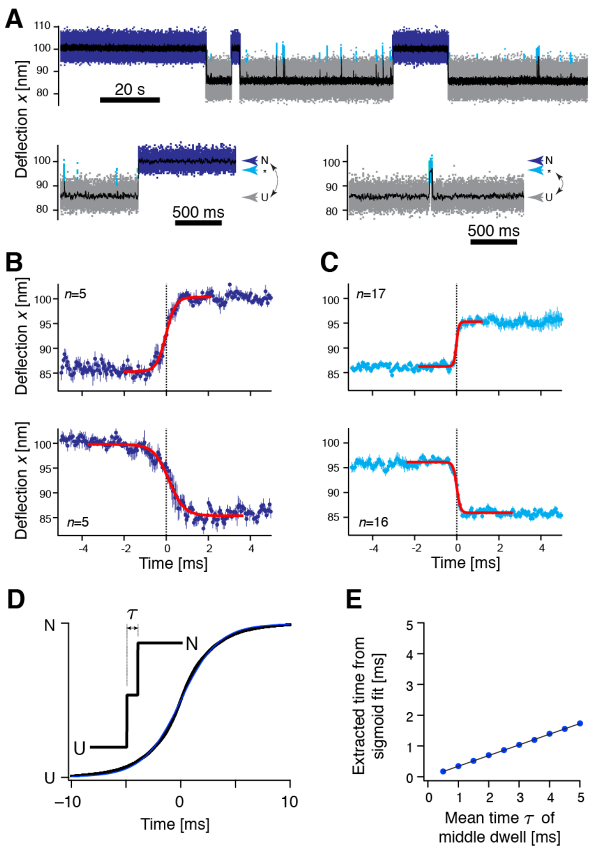

Figure S4. Transition path times from averaging. **(A)** Passive-mode trajectory of ROSS transitioning between N (dark blue) and U (grey) at $F(\mathrm{N}) = 10.6\ \mathrm{pN}$. Transient populations of incomplete folding events with distinct lengths and lifetimes (cyan) are also observed. **(B)** Averaged N-U transitions for folding (top) and unfolding (bottom) after alignment. Continuous red lines are fits to a sigmoidal equation. The corresponding characteristic time of a middle-dwell intermediate is ~900 µs. **(C)** Averaged transitions between U and incomplete folds. The corresponding characteristic time of a middle-dwell intermediate is ~190 µs. The incomplete folding events exhibit a contour length of ≈ 10 nm and are therefore approximately compatible with the length of I_1 (8.3±1.1 nm) but very distinct from N (0 nm) or NL (0.9±1.3 nm). **(D)** Black line: Simulation of averaged transitions with an obligatory intermediate with exponentially distributed time constant (see inset). Blue line: Fit to sigmoidal equation. **(E)** Characteristic timescales from sigmoidal fit vs. mean time of obligatory intermediate for different mean times τ. The slope of this equation is an interconversion factor between the sigmoidal characteristic time and the characteristic time of the obligatory intermediate model.

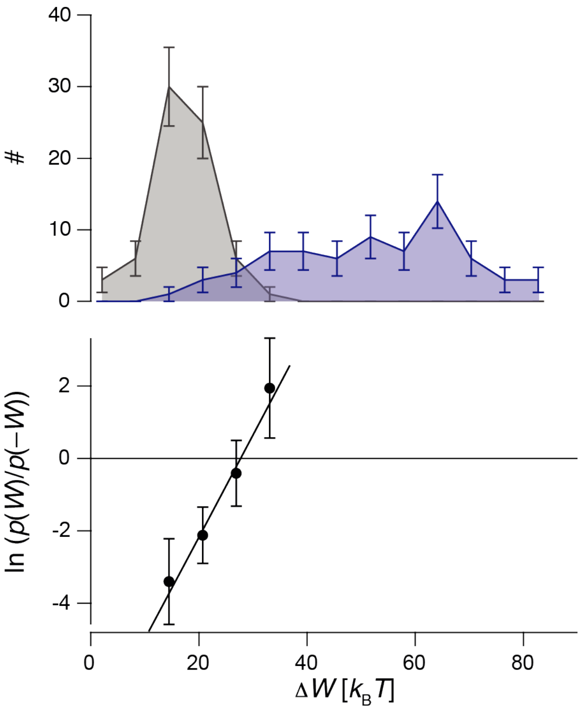

Figure S5. Crooks fluctuation theorem applied to a set of stretch-relax cycles of ROSS at 500 nm/s. Blue: Unnormalized work histogram for unfolding. Grey: Unnormalized work histogram for folding. The resulting free energy difference is –27±2 k_BT.

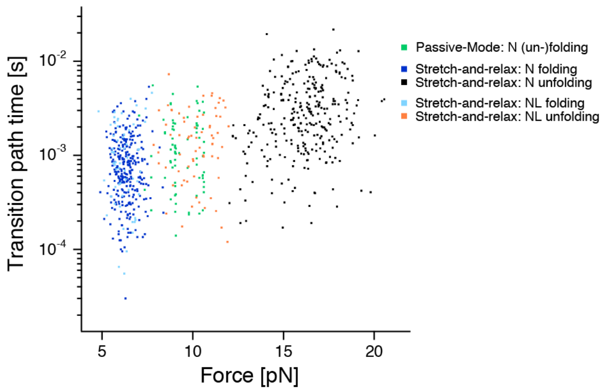

Figure S6. Transition path times of **Fig. 3A**, with color code according to measurement mode and separation of transitions involving either the native (N) or the native-like (NL) state. For passive-mode, only transition path times from N folding and unfolding transitions are shown (green dots, n = 77). Transitions involving NL were not observed in passive-mode within the investigated force range. For unfolding transitions from stretch-relax experiments (500 nm/s), N (black, n = 291) and NL transitions (orange, n = 70) were separated by their unfolding force where unfolding below 12 pN is assigned to NL and unfolding above 12 pN is assigned to N, see also **Fig. 1B.** Further, the unfolding force also determined its preceding refolding transition, i.e., for unfolding forces above 12 pN, the preceding refolding transition is assigned to N (dark blue, n = 286) whereas unfolding forces below 12 pN are the result of refolding into NL (light blue, n = 62), where we assume that there is no direct interchange between N and NL at zero force. Transition path times from refolding transitions out of stretch-relax experiments, which lack a successive unfolding event to probe the nature of refolding, were excluded from our analysis.

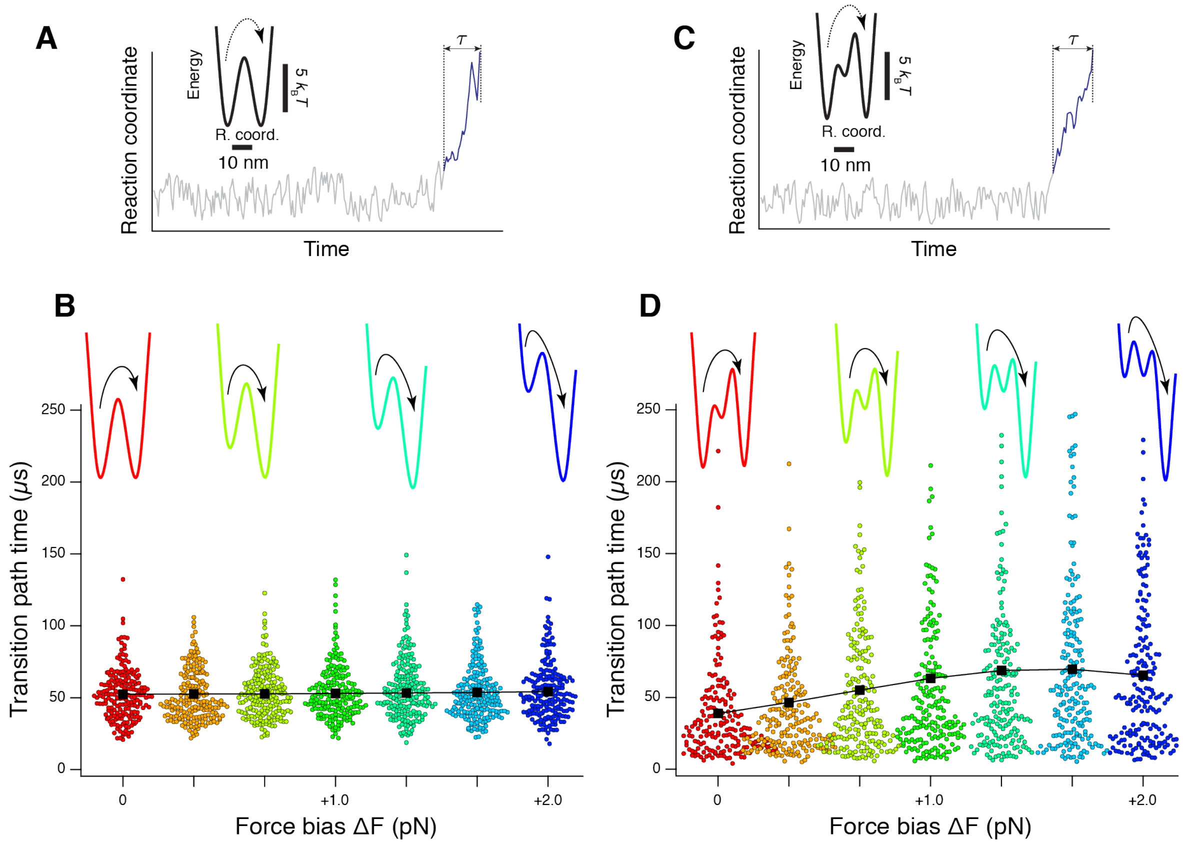

Figure S7. Transition path times of energy landscapes with intermediates and asymmetric barriers have complicated force-dependencies. **(A)** Simulated trajectory (grey) of diffusion in an energy landscape with a simple barrier and no intermediate (inset). TPTs were defined as the time to cross the 14 nm wide barrier. **(B)** Distributions of transition path times from simulations at different force bias for the simple barrier of (A) and $D = 0.2 \times 10^6\ \mathrm{nm^2/s}$. Higher tension was introduced by adding an additional energy term $-\Delta F \cdot x$, where the reference energy landscape shown in (A) was defined as having no force bias ($\Delta F = 0$**).** Black dots and lines indicate the mean of the respective distribution. There is no discernible change of the mean transition path time with increasing force bias. **(C)** Simulated trajectory (grey) of diffusion in an energy landscape with a metastable intermediate and asymmetric barriers (inset). As in (A), TPTs were defined as the time it takes to cross the 14 nm wide barrier. **(D)** Distributions of transition path times from simulations at different force bias for the barrier with metastable intermediate for $D = 10^6\ \mathrm{nm^2/s}$. The diffusion coefficients were chosen to result in similar TPTs for (B) and (D). Black dots and lines indicate the mean of the respective distribution. In contrast to the smooth harmonic barrier, the transition path times increase until a critical force bias where the two transition state barriers have identical height.

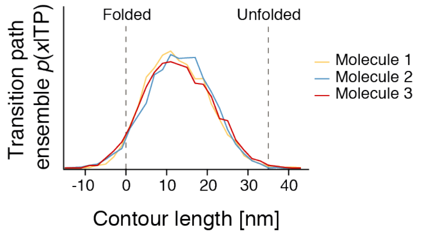

Figure S8. Transition path ensembles from passive mode experiments also show an off-center shape very similar to the shape extracted from constant-velocity experiments (**Fig. 3D**).

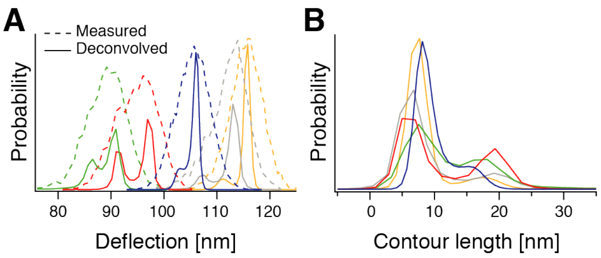

Figure S9. Deconvolution of transition path ensembles extracted from passive-mode experiments. **(A)** Deconvolved probability distributions in deflection space (full lines) and their original transition path ensembles (dashed lines) from different experiments and molecules. Note that normalized probability distributions of measured versus deconvolved distributions have a different scale for reference. **(B)** Deconvolved probability distributions from (A) represented in contour space. The two average peak positions are located at 7.3±0.8 nm and 17.9±1.7 nm which corresponds very well to the positions of the intermediate states I_1 and I_2 derived from WLC-fits to unfolding transitions in constant-velocity experiments (**Fig. 4B**).

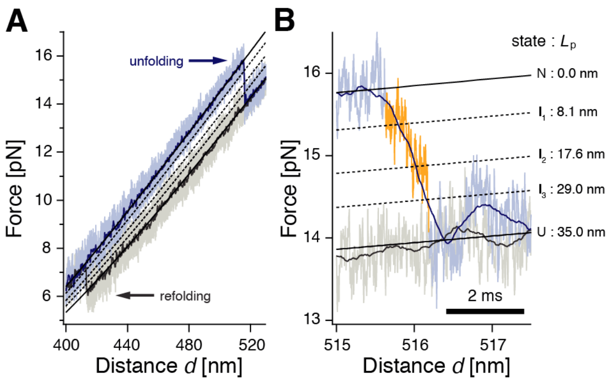

Figure S10. Zoom into the unfolding transition of **Fig. 1A**. **(A)** Force-distance representation of the stretch-relax cycle shown in **Fig. 1A**. Black solid lines are polymer model fits to the folded (N) and unfolded conformations (U). Dashed lines are polymer model fits to local regions within the unfolding transition. **(B)** Zoom into the unfolding transition of (A) by a factor of 50 with respect to distance and, hence, time: at a pulling velocity of 500 nm/s, 1 nm corresponds to 2 ms. The orange part highlights the identified region where ROSS transitions from the native into the unfolded configuration. On the right, the protein contour length gains provided by the polymer model fits are listed.

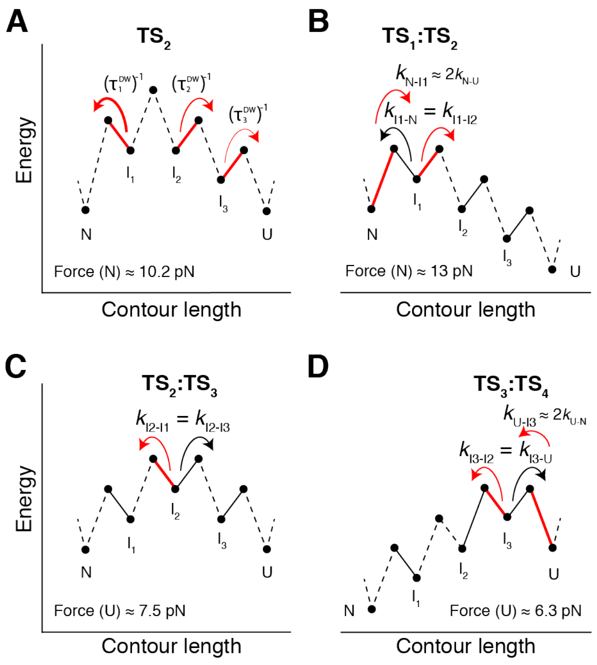

Figure S11. Schematic illustration of the energy landscape reconstruction of ROSS. Red barrier heights are reconstructed, black barrier heights are known from reconstruction in (A) and tilted towards indicated forces acting on the either fully folded (N) or fully unfolded (U) state. **(A)** Energy landscape scenario during equilibrium fluctuations in passive mode where transition state TS_2 prevails and only one single barrier dominates the dwell times within I_1, I_2, and I_3, i.e., the lower one. **(B)** Scenario where a transition state switch between TS_1:TS_2 occurs. Here, when residing in I_1, the rate k_{I1N} over TS_1 must be equal to the rate k_{I1I2} over TS_2. In addition, transition paths that cross from I_1 into I_2 almost never reverse and almost always directly complete the transition into U. Therefore, $2 \cdot k_{\mathrm{NU}}^{\mathrm{N:13pN}} = k_{\mathrm{NI1}}^{\mathrm{N:13pN}}$. **(C)** Energy landscape scenario where a transition state switch between TS_2:TS_3 occurs. Reconstruction of $\Delta G_{\mathrm{I2TS2}}^{\mathrm{U:7.2pN}}$ follows the same line of arguments as in (B). **(D)** Scenario where a transition state switch between TS_3:TS_4 occurs. Reconstructions of $\Delta G_{\mathrm{I3TS3}}^{\mathrm{U:6.3pN}}$ and $\Delta G_{\mathrm{UTS4}}^{\mathrm{U:6.3pN}}$ follow the same principles used in (B).

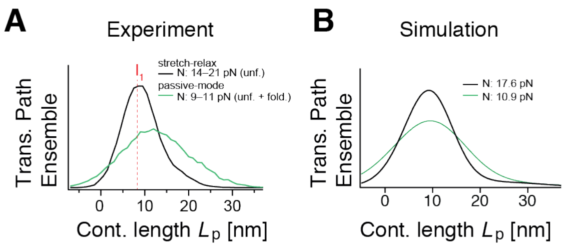

Figure S12. Transition path ensembles from experiments and simulations. **(A)** Comparison between the experimental transition path ensembles from native (un)folding in passive-mode experiments at forces between 9–11 pN and native unfolding in stretch-relax experiments at 14–21 pN. The difference in shape agrees with the prediction from the reconstructed energy landscape (**Fig. 4D**): At higher forces the relative contribution of intermediate I_1 to the overall transition path ensemble increases (cf. **Fig. S7D**). Further, a broader transition path ensemble at lower forces reflects the larger relative contribution of intermediates I_2, I_3 as well as the effect of increased thermal blurring. **(B)** The experimental transition path ensembles agree qualitatively with simulated ensembles based on the barrier profile of **Fig. 4D**. For simulations, we generated 50 trajectories in the D=$10^{6.0}$ nm^2/s barrier profile of **Fig.4D** and simulated experimental broadening by convolution with the PSFs of **Fig. S2**.

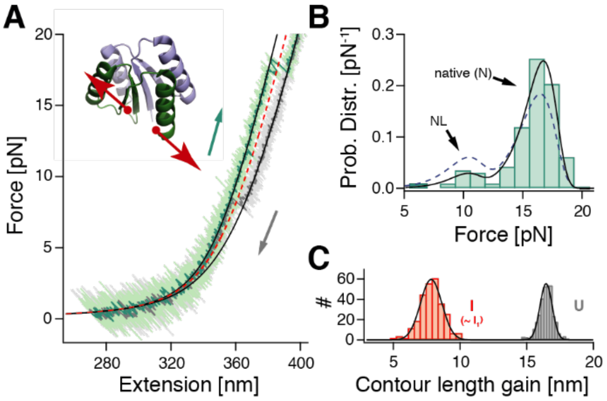

Figure S13. Constant-velocity measurements of the c-terminal pulling variant S49Cc show very similar behavior to ROSS. **(A)** The unfolding pattern of S49Cc shows the same mandatory on-pathway intermediate as ROSS, indicating that both S49Cc and ROSS start unfolding via the c-terminal α-helix. Inset: In S49Cc, force is only applied to the green part. **(B)** For a pulling velocity of 500 nm/s, the unfolding force histogram of S49Cc shows the same two maxima as ROSS (dashed line is the fit to the respective unfolding force distribution of ROSS (**Fig. 1B**)). However, for S49Cc, the fraction of NL unfolding events is significantly smaller ($p < 0.01$, binomial test). Since force is only applied to the c-terminal half of S49Cc, this suggests that non-native interactions between the n- and c-terminal halves of ROSS may be a potential reason for NL-occurrences. **(C)** Histogram of observed contour length gains of S49Cc force-extension trajectories. The observed on-pathway intermediate I of S49Cc corresponds to the intermediate I_1 of ROSS and can be interpreted as the unfolding of the c-terminal α-helix. The length gain for this unfolding is $L_p(\mathrm{I})$ = 8.0±0.9 nm which agrees very well with $L_p(\mathrm{I}_1)$ = 8.3±1.1 nm from ROSS.

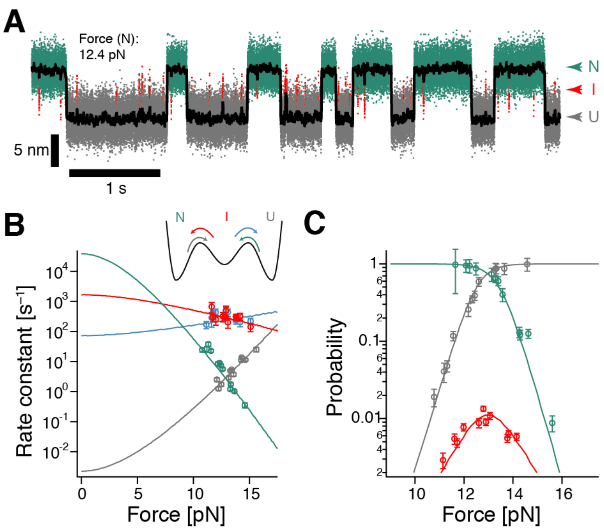

Figure S14. Passive mode measurements allow a more detailed characterization of the on-pathway intermediate I of S49Cc. **(A)** Passive-mode trajectory of S49Cc where the folded state (green) is held at 12.4 pN. HMM analysis identified fast excursions into the intermediate state I (red) from both the folded N (green) and unfolded U (gray) state. **(B)** Chevron plot for extracted transition rates and their respective fits. The inset at the top shows a schematic energy landscape indicating corresponding barrier crossings based on matching colors between fits and arrows. The transition rate fits for transitions from I to N (red) and I to U (blue) intersect at 13 pN with rate constants of about 250 s^{-1}. This yields an effective dwell time of state I of $\tau_I = {}^{1}/_{(k_{IN} + k_{IU})} \approx 2$ ms. **(C)** Probability distribution of N (green), I (red) and U (gray). The maximal probability of being in state I is reached at 13 pN, where the longest dwell time can be expected due to the transition state switch constellation of the two transition states separating I from N and U. The overall folding free energy derived for S49Cc equals $\Delta G_0^{NU} = -21 \pm 2\ k_B T$, where the stability of the intermediate I comprises $\Delta G_0^{NI} = -15 \pm 2\ k_B T$.

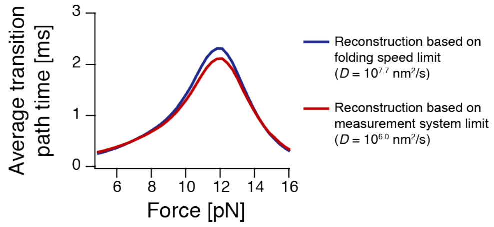

Figure S15. Prediction of the force-dependence of the average transition path time of ROSS based on the two energy landscapes of **Fig. 4D**.

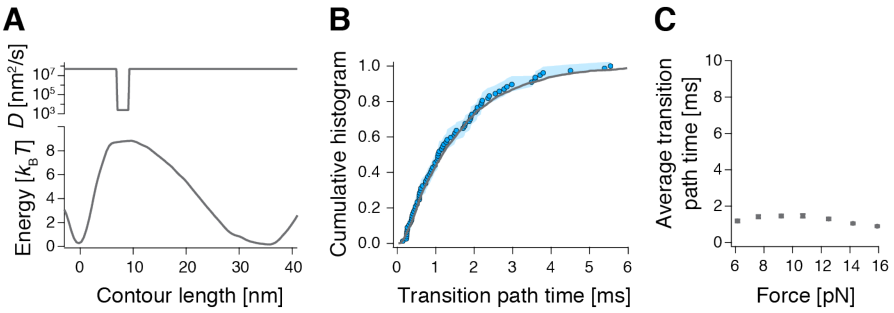

Figure S16. Position-dependent diffusion may also cause a wide distribution of transition path times but fails to explain force-dependent TPTs. **(A)** Deconvolved smooth barrier profile of **Fig. 3B** (bottom) with a position-dependent diffusion coefficient (top) where a stretch with a lower $D=10^{3.4}$ nm^2/s was positioned at the location of I_1. $D=10^{7.7}$ nm^2/s elsewhere. (**B)** Flux-sampling simulations of the cumulative distribution of transition path times from the scenario of (A) (grey line) agrees with experimental data (cyan data points). **(C)** When repeating the simulation in the context of the given diffusion profile, and the energy profile of (A) at different force biases, the average transition path time is largely unaffected, in disagreement with experimental data (cf. **Fig. 3A**).
